# Persistence of Hepatitis C Virus Traces after Spontaneous Resolution of Hepatitis C

**DOI:** 10.1371/journal.pone.0140312

**Published:** 2015-10-16

**Authors:** Annie Y. Chen, Matthew Hoare, Arun N. Shankar, Michael Allison, Graeme J. M. Alexander, Tomasz I. Michalak

**Affiliations:** 1 Molecular Virology and Hepatology Research Group, Faculty of Medicine, Memorial University, St. John’s, Newfoundland and Labrador, Canada; 2 Cancer Research UK Cambridge Institute, Cambridge, United Kingdom; 3 Department of Medicine, University of Cambridge, Cambridge, United Kingdom; Saint Louis University, UNITED STATES

## Abstract

Hepatitis C virus (HCV) frequently causes chronic hepatitis, while spontaneous recovery from infection is infrequent. Persistence of HCV after self-limited (spontaneous) resolution of hepatitis C was rarely investigated. The current study aimed to assess incidence and robustness of HCV persistence after self-resolved hepatitis C in individuals with normal liver enzymes and undetectable virus by conventional tests. Applying high sensitivity HCV RNA detection approaches, we analyzed plasma and peripheral blood mononuclear cells (PBMC) from individuals with previous hepatitis C infection. Parallel plasma and PBMC from 24 such non-viraemic individuals followed for 0.3–14.4 (mean 6.4) years were examined. Additional samples from 9 of them were obtained 4.5–7.2 (mean 5.9) years later. RNA was extracted from 250 μl plasma and, if HCV negative, from ~5 ml after ultracentrifugation, and from *ex vivo* stimulated PBMC. PBMC with evidence of HCV replication from 4 individuals were treated with HCV protease inhibitor, telaprevir. HCV RNA was detected in 14/24 (58.3%) plasma and 11/23 (47.8%) PBMC obtained during the first collection. HCV RNA replicative strand was evident in 7/11 (63.6%) PBMC. Overall, 17/24 (70.8%) individuals carried HCV RNA at mean follow-up of 5.9 years. Samples collected 4.5–7.2 years later revealed HCV in 4/9 (44.4%) plasma and 5/9 (55.5%) PBMC, while 4 (80%) of these 5 PBMC demonstrated virus replicative strand. Overall, 6/9 (66.7%) individuals remained viraemic for up to 20.7 (mean 12.7) years. Telaprevir entirely eliminated HCV replication in the PBMC examined. In conclusion, our results indicate that HCV can persist long after spontaneous resolution of hepatitis C at levels undetectable by current testing. An apparently effective host immune response curtailing hepatitis appears insufficient to completely eliminate the virus. The long-term morbidity of asymptomatic HCV carriage should be examined even in individuals who achieve undetectable HCV by standard testing and their need for treatment should be assessed.

## Introduction

Hepatitis C virus (HCV) is a major cause of chronic liver disease, predisposing to cirrhosis and hepatocellular carcinoma (HCC), affecting around 150 million people [1]. Approximately 15% of those infected undergo spontaneous resolution with loss of HCV RNA using conventional analyses [2]. However, HCV may establish low-level, asymptomatic, persistent infection (occult HCV infection or OCI), which can only be identified using methods with greater sensitivity than those used conventionally [3–6]. This has clinical relevance since trace quantities of HCV may be infectious. Thus, 10–20 virions transmit infection to chimpanzees [7,8], while 20–50 virions establish productive infection in cultured human T lymphocytes [9]. Furthermore, OCI has been identified in individuals negative for HCV RNA by conventional testing who remain asymptomatic [6,10] and following otherwise successful antiviral therapy [3–6,11].

Using nucleic acid amplification assays that detect HCV genomes with sensitivity below 10 copies/mL (or virus genome equivalents, vge) or <2.5 vge/μg RNA (<2 IU/mL), it was recognised that HCV can persist in the circulation at levels up to 100 vge/mL with continued replication in liver and peripheral blood mononuclear cells (PBMC) after sustained virological response (SVR) following IFN-based treatment [3–5,12,13]. Furthermore, plasma and PBMC collected after SVR transmitted infection to a chimpanzee causing high level viraemia and liver injury [14] and also productively infected HCV-prone T cells *in vitro* [9]. It is uncertain if highly potent directly acting agents (DAAs) will be more effective in purging residual HCV.

Sustained persistence of trace HCV has also been reported following spontaneous resolution of HCV, but highly sensitive techniques to detect HCV RNA have been applied in only a few cases [3,15]. To study this further we explored a rare collection of paired plasma and PBMC samples from patients followed up to 20 years after self-limiting HCV infection. Procedures enriching HCV by amplifying viral RNA recovered from larger amounts of plasma and from *ex vivo* mitogen-treated PBMC [3–5,9,13–18], detection of HCV RNA replicative (negative) strand and compartmentalized virus variants [3,4,9,13–19] and treatment of PBMC replicating HCV with a DDA [20] were applied to uncover the characteristics and robustness of HCV persistence. In parallel, the study aimed to assess the frequency of OCI incidence in individuals after a self-resolved episode of hepatitis C who repeatedly displayed normal liver enzyme levels and undetectable virus by conventional testing.

## Materials and Methods

### Ethic Statement

The study was approved by the Research Ethics committee of Addenbrooke’s Hospital and performed in accordance with the Declaration of Helsinki. The samples were collected after signing written informed consent.

### Patients and samples

Paired plasma and PBMC samples were collected from 24 randomly selected, asymptomatic individuals (17 men and 7 women; aged 25 to 73 years) with spontaneous resolution of HCV, as established using recognised clinical, biochemical and virological criteria (Table 1). Samples for the current study were obtained a mean 6.4 years (range 0.3–14.4) from first clinical review. Additional paired plasma and PBMC samples were collected from 9 individuals at a mean 5.9 years after first collection (range 4.5–7.2), *i*.*e*., a mean 12.7 years total follow-up (range 5.5 to 20.7) (Table 1). 22/24 described risk factors for exposure to HCV, including injecting drug use (IDU) (n = 21) or multiple blood product transfusions (n = 1) (Table 1). At first sample collection, 21/24 had normal serum alanine aminotransferase (ALT); 3/24 had minimal elevation of ALT. Total bilirubin level was normal in all. One with membranous glomerulonephritis had an abnormal prothrombin time (PT) due to anti-coagulation with warfarin. Of 8 who underwent liver biopsy, 4 had stage 1/6 fibrosis and one each had fibrosis stage 2, 3 and 5 (Table 1). At the second sample collection, biochemical tests remained normal, except a borderline ALT in one male and the PT remained elevated in one. At that time, fibroscan showed normal liver stiffness in 8, while one male had stage 2 fibrosis (Table 1). HCV RNA was undetectable during the entire follow-up as measured by a conventional assay (Artus HCV QS-RGQ Kit, version 1; sensitivity 20 IU/ml or 24.2 copies/ml; Qiagen Gnbh, Hilden, Germany) at approximately yearly intervals (Table 1). All patients were anti-HCV at both sample collections (EIA; Abbott Diagnostics, Maidenhead, United Kingdom) (Table 1). At first clinic review 13/24 were anti-HBc positive and 6 were anti-HBs positive. None was reactive for antibody to human immunodeficiency virus type-1.

**Table 1:** Clinical Characteristics and Time of Sample Collection from Individuals with Spontaneous Resolution of Hepatitis C.

				First Sample Collection						Second Sample Collection							
Case (n)	Age (Years)	Sex (F/M)	Route Of Infection	Follow-up From First Seen (Years)	ALT (0–50 U/L)	Total Bilirubin (0–17 μ/L	PT (9.8–12.6 sec)	Serum HCV RNA (Clinical Assay)*	HCV Genotype	Liver Fibrosis (Stage)	Time From First Collection (Years)	ALT (0–50 U/L	Total Bilirubin (0–17 μM)	PT (9.8–12.6 Sec)	Serum HCV RNA (Clinical Assay)*	Anti- HCV	Liver Fibroscan (kPa)
1	38	M	Unknown	1.8	27	8	13.3	NEG	3	NT							
2	45	F	IDU	4.8	16	8	11.9	NEG	NA	NT							
3	46	M	IDU	1.4	60	7	12.5	NEG	1a	NT							
4	46	M	IDU	0.3	21	13	12.6	NEG	1b	NT	5.2	32	11	10.2	NEG	NA	3.8
5	32	F	IDU	9.5	29	7	12.9	NEG	1	NT							
6	25	M	IDU	0.7	25	13	12.5	NEG	NA	NT							
7	73	F	Blood tx	3.3	22	10	13.2	NEG	NA	NT	5.5	20	10	13.2	NEG	POS	4
8	45	M	IDU	10.3	23	5	11.1	NEG	1a	1							
9	40	M	IDU	11.7	31	6	10.3	NEG	NA	0							
10^‡^	70	M	IDU	2.3	25	7	45	NEG	1a	NT	6.5	18	7	35	NEG	POS	5.7
11	57	M	IDU	10.4	39	10	12.9	NEG	1a	NT							
12	49	M	IDU	10.3	16	2	12.2	NEG	1^§^	1							
13	50	M	IDU	4.3	36	7	10.3	NEG	1b	NT							
14	47	F	IDU	2.9	15	5	10.1	NEG	2a	NT	4.5	20	5	10.1	NEG	POS	3.1
15	46	F	IDU	11	20	5	12.2	NEG	3a	NT	5.1	28	5	12.2	NEG	POS	3.2
16	52	F	IDU	4.8	41	6	12.4	NEG	1b	NT	7.2	28	6	12.4	NEG	POS	5.8
17	47	M	IDU	14.4	21	7	11.8	NEG	NA	1	6.3	31	11	10.2	NEG	POS	3.8
18	41	M	IDU	9.8	9	5	13.8	NEG	NA	2							
19	55	M	Unknown	2.5	37	4	11	NEG	1	5							
20	49	F	IDU	14.3	15	8	11.8	NEG	1b	NT	6.3	24	8	11.8	NEG	POS	4
21	27	M	IDU	2.8	51	7	11.9	NEG	1	NT							
22	44	M	IDU	9.1	8	5	12.8	NEG	1a	3							
23	43	M	IDU	2.9	25	6	12.9	NEG	1	NT							
24^†^	53	M	IDU	7.4	50	7	10.7	NEG	1b	1	6.7	50	7	12.8	NEG	POS	10.7
Mean or Number from Total	46.7			6.4	27.6	7	12	0/24		1.8	5.9	27.9	7.8	11.6	0/9	8/8	4.9

ALT, alanine aminotransferase; PT, prothrombin time; kPA, kilopescals; M, male; F, female; POS, positive; NEG, negative; NA, not available; NT, not tested; IDU, Intravenous drug user; Blood tx, blood transfusion,

*Assay sensitivity 20 IU/m.

^†^Only plasma obtained during the first collection examined

^‡^Patient with membranous glomerulonephritis.

^§^Assessed by clinical test.

PBMCs were isolated from 50–60 ml of whole blood by Ficoll-Hypaque (Pharmacia Biotech, Quebec, Canada) density gradient centrifugation and stored in liquid nitrogen [3,13]. Plasma was stored at -80°C.

### Stimulation of PBMC ex vivo

Previous investigations of low-level (occult) infection with woodchuck hepatitis virus (WHV) and HBV [21,22] or HCV [3,9,13,15,16] demonstrated that *ex vivo* mitogen stimulation of lymphoid cells augments virus replication allowing detection in cells thought to be virus non-reactive. PBMC were cultured for 72 h with 5 μg/ml phytohemagglutinin (PHA; a T cell stimulating mitogen) (Sigma-Aldrich, Mississauga, Ontario, Canada) and if they remained HCV RNA negative, with C5, a cocktail containing 5 μg/ml PHA, 5 μg/ml pokeweed mitogen (a B and T cell stimulating lectin) (ICN Biomedicals, Irvine, CA), 20 U/ml interleukin-2 (IL-2; a T cell growth supporting cytokine) (Roche Molecular Diagnostics, Pleasanton, CA) and 1 ng/ml IL-4 (a B-cell growth supporting cytokine) (Roche Molecular Duiagnostics). Superiority of C5 over PHA in regard to up-regulation of HCV replication and HCV RNA expression has been reported [16]. PBMC were cultured as previously reported [13,16].

### RNA extraction and transcription

Total RNA was extracted with Trizol LS reagent (Invitrogen) from 250 μl plasma or 5 ml plasma following ultracentrifugation at 273,800 x *g* for 22 h at 4°C [13]. RNA from 5 x 10^6^ to 1 x 10^7^ native or stimulated PBMC was extracted with Trizol (Invitrogen Life Technologies, Burlington, Ontario, Canada). Each series of RNA extractions were done in parallel with mock extraction using sterile water (contamination control), serum or PBMC from a healthy donor (negative control), and serum or PBMC from a patient positive for plasma HCV RNA (positive control). RNA from cells (2 to 4 μg) and all RNA obtained from plasma were transcribed to cDNA [3].

### Detection of HCV RNA positive and negative strands

For detection of HCV RNA positive (replicative) strand, reverse transcriptase-polymerase chain reaction (RT-PCR) was applied using primers specific for the HCV 5ˋ-untranslated region (5ˋ-UTR), cycling conditions, quantification standards and controls reported [3,13,18]. Depending upon sample availability, amplification with primers specific for HCV E1/E2 region, encompassing hypervariable region 1 (HVR1), was also performed (S1 Table). Detection of HCV RNA in OCI normally requires two rounds of PCR, stringent precautions and appropriate controls at each step of RNA and cDNA preparation and PCR amplification. In all instances, signal specificity was confirmed by nucleic acid hybridization (NAH) using ^32^P-labeled recombinant HCV 5ˋ-UTR-E2 fragment as a probe [3]. Sensitivity of the RT-PCR/NAH assays with either 5ˋ-UTR or E1/E2 region-specific primers was ≤10 vge/mL or ≤2.5 vge/μg RNA. HCV RNA negative (replicative) strand was detected by RT-PCR/NAH, as described in detail before [3,20]. This assay detects ~10^2^ copies of the correct (negative) strand, while identifying ≥10^6^ copies of the positive strand. Specificity of amplicons and validity of the controls was confirmed by NAH. A number of negative and contamination controls were included in each analysis [3,8,13,18].

### Cloning, sequencing and HCV sequence analyses

To assess possible sequence variation and compartmentalization of HCV, 5ˋ-UTR and, when available, E1/E2 amplicons were cloned using the TOPO-TA cloning system (Invitrogen). The highly conserved 5ˋ-UTR was chosen because it allows for reliable identification of most unwavering sequence variants. Up to 10 randomly selected clones were analyzed from each PCR product derived from plasma and PBMC collected from 2 individuals (15-46/F and 20-49/F) followed for 16.1 and 20.6 years, respectively. The clones were sequenced in both directions using universal forward and reverse M13 primers and the ABI-Prism 7000 Sequence Detection System (Applied Biosystems, Streetsville, Ontario, Canada).

The HCV genotype was known for only one patient prior to this study (Table 1), so the resulting 5'-UTR sequences were aligned with help of Sequencher software version 4.7 (Gene Codes Corporation, Ann Arbor, MI). The HCV subgenotype sequences AF011751 and M62321 for genotype 1a, D90208 and D11168 for genotype 1b, AF169005 for genotype 2a, AF238486 for genotype 2b, D17763 and NC009824 for genotype 3a, and D11443 and D49374 for 3b from GenBank served as references. The phylogenetic relationships of the variant 5ˋ-UTR sequences identified in plasma and PBMC of 15-46/F and 20-49/F were analyzed with DNA-DIST in the PHYLIP (version 3) software package and trees constructed using NEIGHBOR program (University of Washington, Seattle, WA).

### Treatment of HCV-infected PBMC with telaprevir

Telaprevir (TLP) (Vertex Pharmaceuticals, Cambridge, MA), an HCV-specific protease inhibitor [23], inhibits HCV replication effectively in *de novo* HCV-infected T cell lines [20]. An established protocol was applied to determine whether HCV replication in PBMC is suppressed by treatment with TLP. Briefly, ~1x10^7^ PBMC acquired from 14-47/F, 15-46/F and 20-49/F at 14.7 years follow up (range 7.4–20.6) (Table 1), reactive for HCV RNA positive and negative strands, were cultured in duplicate for 72 h in 5 ml of complete RPMI medium with 5 μg/ml PHA or C5 and 4 μM TLP in 0.5% DMSO. The same PBMC cultured in duplicate with 0.5% DMSO alone served as controls. In the preceding experiments, it was confirmed that TLP at concentrations equal to or below 4 μM in 0.5% DMSO was not toxic to normal human PBMC, as for Molt4 T cells [20]. Expression of HCV RNA positive and negative strands was evaluated in TLP-treated and control PBMC as indicated above.

### Statistical analysis

Results were analyzed by Chi-Square test using SPSS Statistics software version 19.0 (IBM, Armonk, NY). Differences between sample groups were considered to be significant when *P* values were below or equal to 0.05.

## Results

### Detection of HCV RNA in plasma after spontaneous resolution

Paired plasma and PBMC samples were collected from 24 randomly selected, asymptomatic individuals (17 men and 7 women; aged 25 to 73 years) with spontaneous resolution of HCV, as established using recognised clinical, biochemical and virological criteria (Table 1). Samples were obtained a mean 6.4 years (range 0.3–14.4) from first clinical review. Additional paired plasma and PBMC samples were collected from 9 individuals at a mean 5.9 years after first collection (range 4.5–7.2), *i*.*e*., a mean 12.7 years total follow-up (range 5.5 to 20.7) (Table 1).

**Fig 1:**
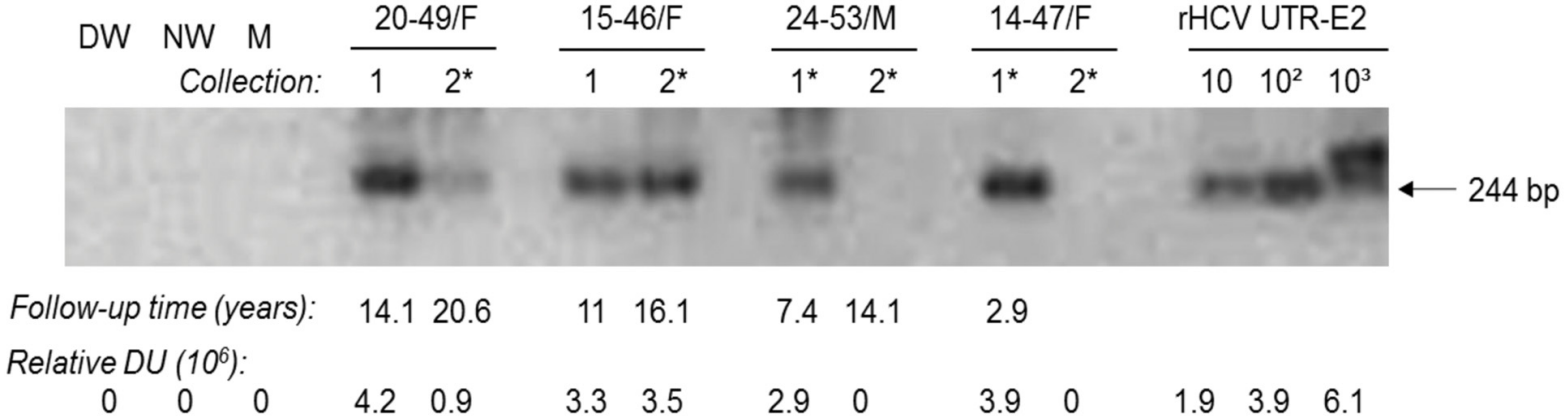
Detection of HCV RNA in plasma obtained at two separate collections from individuals with a past episode of spontaneously resolved hepatitis C. Total RNA extracted from 250 μl or a pellet recovered after ultracentrifugation of ~5 ml plasma (*) acquired during the first (1) and the second (2) sample collections were amplified with 5'-UTR specific primers and amplicon specificity verified by NAH. Serial 10-fold dilutions of recombinant HCV 5'-UTR-E2 (rHCV UTR-E2) fragment carrying indicated copy numbers/reaction were used as positive and specificity controls. Water amplified in direct (DW) and nested (NW) reactions and a mock (M) extraction served as contamination controls. Positive signals showed the expected 244-bp oligonucleotide fragments. Numbers under the panels represent follow-up time in years after resolution of hepatitis C (upper line) and relative densitometric units (DU) given by hybridization signals (lower line).

Plasma samples obtained during the first (n = 24) or the second (n = 9) collections were uniformly HCV RNA negative by standard clinical testing (Table 1). However, when the same samples were re-analyzed using RT-PCR/NAH assay, 58.3% (14/24) and 44.4% (4/9) were HCV RNA reactive at mean follow-up 6.4 and 12.7 years (ranges 0.3 to 14.4 and 5.5 to 20.7 respectively) (Table 2 and Fig 1). The majority of HCV RNA reactive samples from the first collection (11/24; 45.8%) were positive when RNA from 250 μl plasma was analyzed. The remaining (3/14; 21.4%) were HCV RNA positive when pellets from ~5 ml of plasma were tested (Table 2). Inversely, 3 of 4 (75%) plasma samples from the second collection were HCV RNA positive after ultracentrifugation (Table 2). Overall, 17 of 24 (70.8%) individuals carried HCV in either one or two plasma samples collected.

**Table 2:** Total Numbers of Plasma and PBMC Samples from individuals with Spontaneous Resolution of Hepatitis C And Results on HCV Detection.

	Plasma				PBMC							
		HCV RNA+ Positivity				HCV RNA+ Positivity				HCV RNA- Positivity		
Sample Collection	Total Sample Tested	250 μl	5 ml	Total Positivity (%)	Total Samples Tested	PHA-Treated	C5-Treated	Total Positivity (%)	Samples Tested*	PHA- Treated	C5Treated	Total Positivity (%)
First	24	11	3	14/24	23	11	NA	11/23	11	11	NA	7/11
				(58.3%)				(47.8%)				(63.6%)
Second	9	1	3	4/9	9	2	3	5/9	5	2	3	45
				(44.4%)				(55.6%)				(80%)

PBMC, peripheral blood mononuclear cells; HCV RNA+, HCV RNA positive strand; HCV RNA-, HCV RNA negative strand; PHA, phytohemagglutinin; C5, immune cell stimulating cocktail (see Materials and Methods); NA, not applicable.

*Tested only when the sample found HCV RNA positive strand reactive.

### Expression of HCV genome in PBMC years after spontaneous resolution

Because of the expected very low level HCV RNA and limited availability of PBMC, cells from the first collection were stimulated *ex vivo* with PHA without prior testing of untreated cells, as in previous studies [3,13]. In regard to PBMC acquired during the second collection, they were first cultured with PHA and, if HCV RNA positive strand was undetectable, their second sample were cultured with C5. As shown in Table 2, 11 of 23 (47.8%) PHA-treated PBMC from the first collection showed HCV RNA positive strand reactivity. Also, 2 of 9 (22.2%) PBMC from the second collection were HCV RNA positive after PHA stimulation (Table 2). Among the remaining 7 samples from the second collection, 3 additional displayed HCV RNA after culture in the presence of C5. In total, 5/9 (55.5%) PBMC samples collected at mean follow-up of 12.7 years (range 5.5 to 20.6 years) were HCV RNA reactive (Table 2). In total, 13 of 23 (56.5%) individuals who provided either one or two PBMC samples were HCV RNA positive. Considering the results from analyses of both plasma and PBMC obtained during both collections, 70.8% (17/24) of individuals were HCV RNA positive despite being negative by conventional assays.

### HCV replication in PBMC after spontaneous resolution

**Fig 2:**
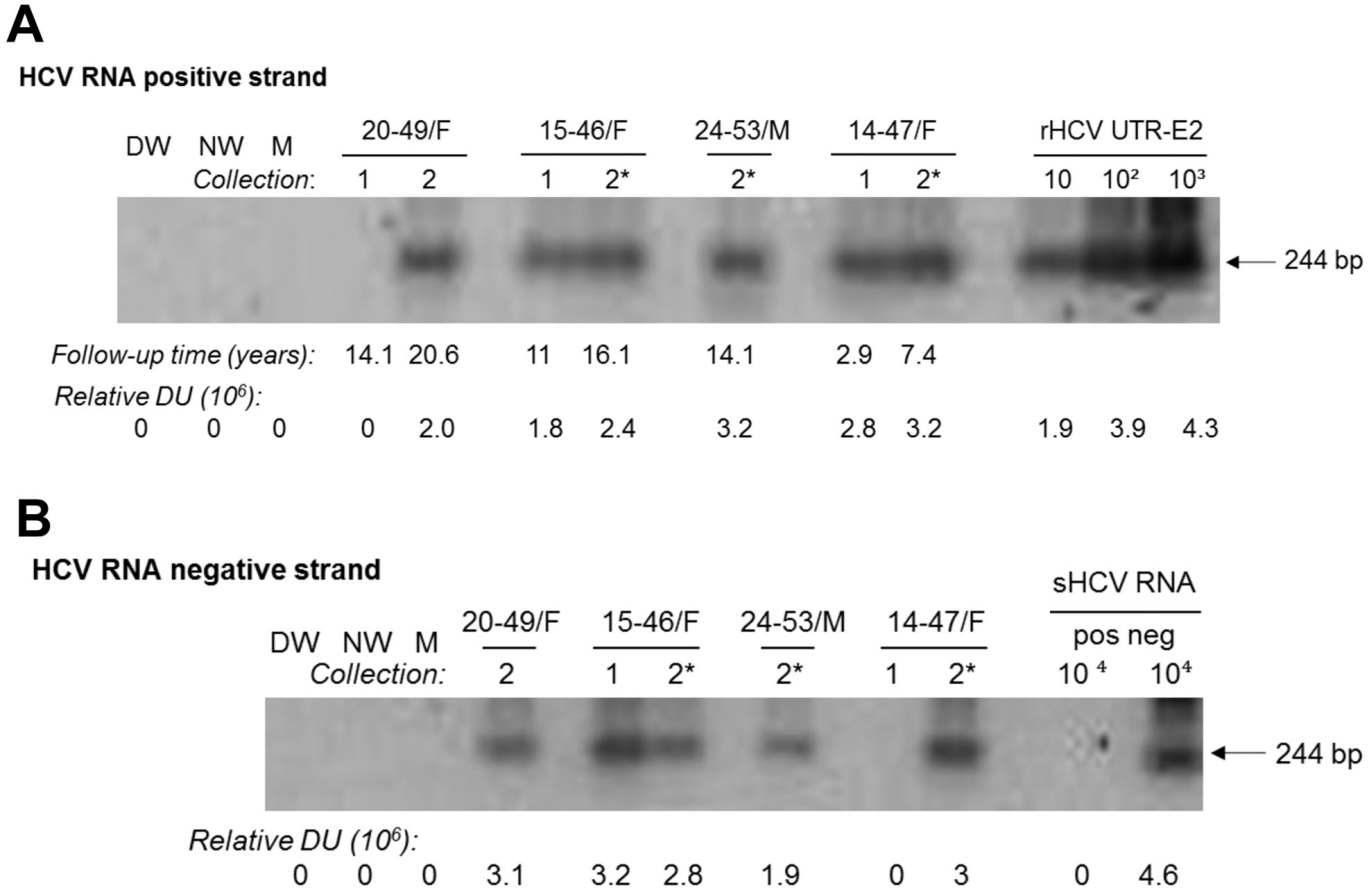
Expression of HCV RNA positive and negative (replicative) strands in PBMC samples obtained at two collections approximately 5 years apart from individuals with self-resolved hepatitis C. (A) HCV RNA positive strand identification using 2 μg of total RNA extracted from PBMC treated *ex vivo* with PHA or C5 (*). (B) Detection of HCV RNA negative strand in HCV RNA positive strand reactive PBMC shown in A. Samples were obtained during the first (1) or the second (2) collection at follow-up time (years) indicated under panel A. As positive controls for HCV RNA positive strand detection, serial 10-fold dilutions of rHCV UTR-E2 carrying indicated copy numbers/reaction were used (panel A). For HCV RNA negative strand detection, synthetic HCV RNA positive strand (sHCV RNA pos) and HCV synthetic RNA negative strand (sHCV RNA neg) at 10^4^ copies/reaction were used as positive and specificity controls, respectively (panel B). Water amplified in direct (DW) and nested (NW) reactions and a mock (M) extraction served as contamination controls. Positive signals demonstrated the expected 244-bp oligonucleotide fragments. Numbers under panels marked as relative DU represent relative densitometric units (DU) given by hybridization signals.

PBMC found to be HCV RNA positive strand reactive were examined for expression of the HCV RNA negative (replicative) strand. This replication intermediate occurs at a lower copy number per cell than the positive strand and the assay detecting the negative strand is 10 to 100-fold less sensitive than that for the positive strand identification [3,20], so HCV RNA negative strand was tested only in PBMC found reactive for the positive strand. Thus, this replication intermediate was detected in 11 out of 16 (68.7%) PBMC investigated (Table 2 and Fig 2). Among the negative strand reactive PBMC samples, 7 (7/11; 63.6%) were obtained during the first collection (mean follow-up 5.1 years) and 4 (4/5; 80%) at the second (mean follow-up 14.5 years) (Table 2).

### Detection of HCV in paired plasma and PBMC samples collected years apart after spontaneous resolution

Examination of HCV expression in paired plasma and PBMC samples collected at two occasions 4.5 to 7.2 years apart was possible in 9 patients. Considering HCV RNA detection in either 250-μl or ~5 mL plasma and in PBMC obtained at both collections (Table 3), two individuals demonstrated continued presence of low-level HCV. Hence, one (15-46/F) showed HCV in both plasma and PBMC samples at 11 and 16.1 years follow-up, while another (20-49/F) displayed virus reactivity in both plasma samples collected after 14.3 and 20.6 years and in PBMC obtained at first collection. Notably, all positive strand reactive PBMC samples (n = 3) from both cases also expressed virus RNA replicative strand (Table 3 and Fig 2). In another two subjects (14-47/F and 24-53/M), although the first plasma collected carried HCV RNA, plasma obtained 2.9 and 7.4 years later were virus nonreactive. Nonetheless, the final PBMC samples obtained after 7.4 and 14.1 years of follow-up, respectively, were HCV RNA positive and importantly, negative strand reactive (Table 3 and Fig 2). In two others (4-46/M and 16-52/F), HCV RNA was detected in plasma acquired during the second but not the first collection, whereas PBMC obtained from either the first or the second collection were HCV RNA positive strand reactive but negative for the replicative strand (Table 3). In 10-70/M followed for 8.8 years, HCV was undetectable in both plasma samples, while the first PBMC sample but not the second was virus positive in the absence of the negative strand detection. Finally, paired plasma and PBMC samples collected at both bleedings from 7-73/F and 17-47/M were HCV nonreactive. These individuals were followed for 8.8 and 20.7 years, respectively, indicating that they were consistently HCV negative (Table 3). Overall, considering HCV detection in the last either plasma or PBMC samples available for analysis, the majority (6/9; 66.7%) of the cases demonstrated persistence of HCV traces lasting up to 20.7 (mean 12.7) years, while 3 (33.3%) individuals were either consistently HCV negative or eliminated virus after the first sample collection.

**Table 3:** Detection of HCV in Two Sequential Paired Plasma and PBMC.

		First Sample Collections				Second Sample Collections					
		HCV RNA + Strand				HCV RNA + Strand					
		Plasma					Plasma		PBMC		
Patient Age/Sex	Genotype	250 μl	~5 ml	PBMC PHA-Treated	HCV RNA-Strand PBMC	Time from First Collection (Years)	250 μl	~5 ml	PHA-Treated	CS-Treated	HCV RNA-Strand PBMC
4-6/M	1b	NEG	NEG	NEG	NT	5.2	NEG	POS	POS	NT	NEG
7-73/F	NT	NEG	NEG	NEG	NT	5.5	NEG	NEG	NEG	NEG	NT
10-70/M	1a	NEG	NEG	POS	NEG	6.5	NEG	NEG	NEG	NEG	NT
14-47/F	2a	NEG	POS	POS	NEG	4.5	NEG	NEG	NEG	POS	POS
15-46/F	3a	POS	NT	POS	POS	5.1	NEG	POS	NEG	POS	POS
16-52/F	1b	NEG	NEG	POS	NEG	7.2	POS	NT	NEG	NEG	NT
17-47/M	NT	NEG	NEG	NEG	NT	6.3	NEG	NEG	NEG	NEG	NT
20-49/F	1b	POS	NT	NEG	NT	6.3	NEG	POS	POS	NT	POS
24-53/M	1b	NEG	POS	NA	NA	6.7	NEG	NEG	NEG	POS	POS
Mean or Number From Total		2/9	2/7	4/8	1/4	5/9	1/9	3/8	2/9	3/7	4/5

PBMC, peripheral blood mononuclear cells; HCV RNA+, HCV RNA positive strand; HCV RNA-, HCV RNA negative strand; PHA, phytohemagglutinin; C5, immune cell stimulating cocktail (see Materials and Methods); POS, positive; NEG, negative; NT, not tested; NA, not available.

### Minor variations in sequence of HCV persisting after spontaneous resolution

**Fig 3:**
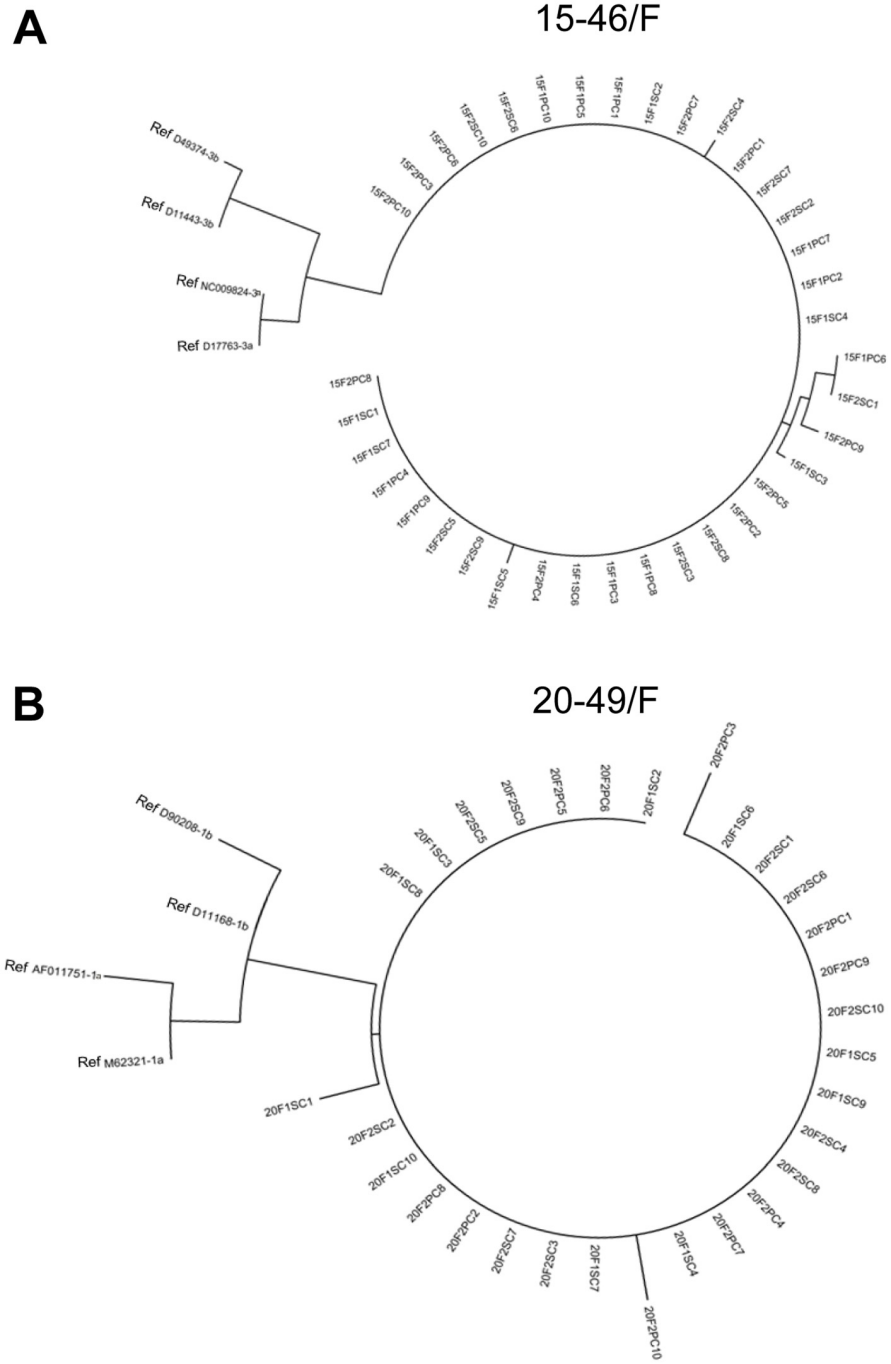
Phylogenetic analysis of point mutations identified in the 244-bp HCV 5’-UTR fragment from plasma and PBMC of 15-46/F and 20-49/F patients with 7.4-year and 20.6-year observation periods, respectively, after spontaneous recovery from hepatitis C. (A) HCV sequences identified in plasma and PBMCs of 15-46/F. (B) HCV sequences found in plasma and PBMCs of 20-49/F. The numbers 15 and 20 identify patient 15-46/F and 20-49/F, respectively. The numbers 1 and 2 stand for the first or the second collection of samples. The variants from plasma are marked with S and those from PBMC with P. The numbers 1–10 indicate individual clones. The nucleotide sequences of genotypes 3a and 3b for 15-46/F and 1a and 1b for 20-49/F serving as references are marked as Ref.

A significant effort was made to recognize sequence stability and potential compartmentalization of variants of HCV persisting after spontaneous resolution. Clones of 5’-UTR 244-bp amplicons derived from plasma and PBMC of 15-46/F (subgenotype 3a) collected at two occasions 5.1-year apart and from 20-49/F (subgenotype 1b) obtained at 14.3 and 20.6 years of follow-up were sequenced (see Table 1). The resulting sequences were compared to relevant HCV sub-genotypes from GenBank and to each other taking the sequence identified in plasma from the first collection as base line (Table 4). Analysis showed three types of point mutations. The first was identified in all clones derived from either plasma or PBMC obtained at both collections, which reflected variant sequences of a given sub-genotype not reported before, as a search of the published data deposited in GenBank implied, *i*.*e*., A120C and C203T in 15-46/F, and G110A and G295C in 20-49/F (Table 4 and Fig 3). The second type were point mutations, which occurred sporadically in one or two clones from plasma or PBMC obtained at both collections, *i*.*e*., A134G, C209T and A223C in 15-46/F, and 127insC in 20-49/F (Table 4 and Fig 3). The third type were very rare point mutations unique to a given compartment, *i*.*e*., plasma or PBMC, which did not have equivalence in another compartment and occurred only at one time point, *i*.*e*., C126A and T212C in 15-46/F, and A96C, C204T and G241A in 20-49/F (Table 4). In general, the analysis verified that HCV persisted in both plasma and PBMC, and suggested that there was only a slight tendency towards variant compartmentalization and replication progressed at a very low rate, as inferred from the rare occurrence of point mutations unique to either plasma or PBMC.

**Table 4:** Single-Nucleotide Polymorphisms in the HCV 5’-UTR Sequence from Plasma and PBMC Obtained at Two Collections Done More than 5 Years Apart from Patients after Spontaneous Resolution of Hepatitis C.

Patient Age/Sex	HCV Genotype	Collection (Years of Follow-up)	Sample	Point Mutations						
				**A120C**	**C126A**	**A134G**	**C203T**	**C209T**	**T212C**	**A223C**
15-26/F	3a	First (11)	Plasma	7/7	ND	1/7	7/7	ND	1/7	ND
			PBMC	10/10	ND	ND	10/10	1/10	ND	1/10
		Second (16.1)	Plasma	10/10	1/10	ND	10/10	1/10	ND	1/10
			PBMC	10/10	ND	1/10	10/10	1/10	ND	ND
				**A96C**	**G110A**	**127*ins*C**	**C204T**	**G241A**	**G295C**	
20–49	1b	First (14.3)	Plasma	ND	10/10	2/10	1/10	ND	10/10	
		Second (20.6)	Plasma	ND	10/10	1/10	ND	ND	10/10	
			PBMC	1/10	10/10	ND	ND	1/10	10/10	

The results are presented as numbers of clones in which a given mutation was identified per total number of clones tested. The designation of the nucleotide position based on the prototype 3a and 1b subgenotype sequences D1773 and D11168, respectively, from GenBank. ND, not detected; NA, not available

**Fig 4:**
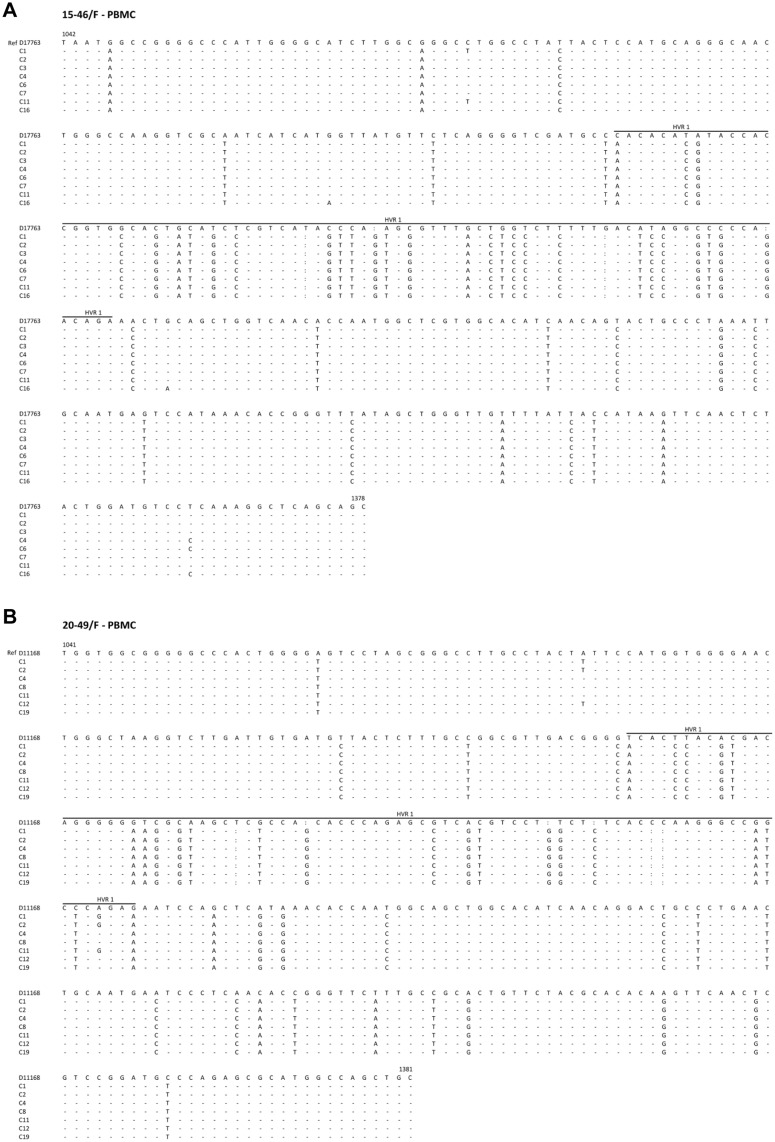
Single-nucleotide polymorphisms in the HCV E1/E2 330-bp fragment derived from PBMC of two individuals with long-term follow-up after spontaneously resolved hepatitis C. (A) Sequence identified in PBMC after 7.4 years of observation of 15-46/F carrying HCV genotype 3a. (B) Sequence from PBMC of 20-49/F followed for 20.6 years carrying HCV genotype 1b. Seven or eight randomly selected clones were sequenced bi-directionally and their sequences compared to respective subgenotype D17763 and D11168 sequences from GenBank. The boundaries of the hypervariable region 1 (HVR1) are marked by a line. Numbers and the beginning and at the end of each reference sequence, marked as Ref, indicate nucleotide positions. Nucleotides identical to those in the reference sequence are shown as dots, different as letters, and deleted as colons.

Attempts to identify variants in the E1/E2 sequence encoding HVR1 were only partially successful because of limited material. Nonetheless, PBMC acquired after 16.1 and 20.6 years of follow-up of 15-46/F and 20-49/F, respectively, showed multiple point mutations common to all clones sequenced when compared to respective 3a or 1b references (Fig 4). The majority of these mutations occurred, as expected, in HVR1 and we believe not reported before. There were also very few (2–4) unique point mutations identified in one to 3 of 8 (15-46/F) or 7 (20-49/F) clones sequenced (Fig 4). This was consistent with a high degree of sequence homogeneity within and beyond HVR1 in PBMC analyzed.

The 5’-UTR and E1/E2 sequences identified in this study have been deposited in GenBank under accession numbers: KP861251-KP861287 and KP861318-KP861325 for 15-46/F, and KP861288-KP861317 and KP861326-KP861332 for 20-49/F.

### Inhibition of persisting HCV replication by telaprevir

**Fig 5:**
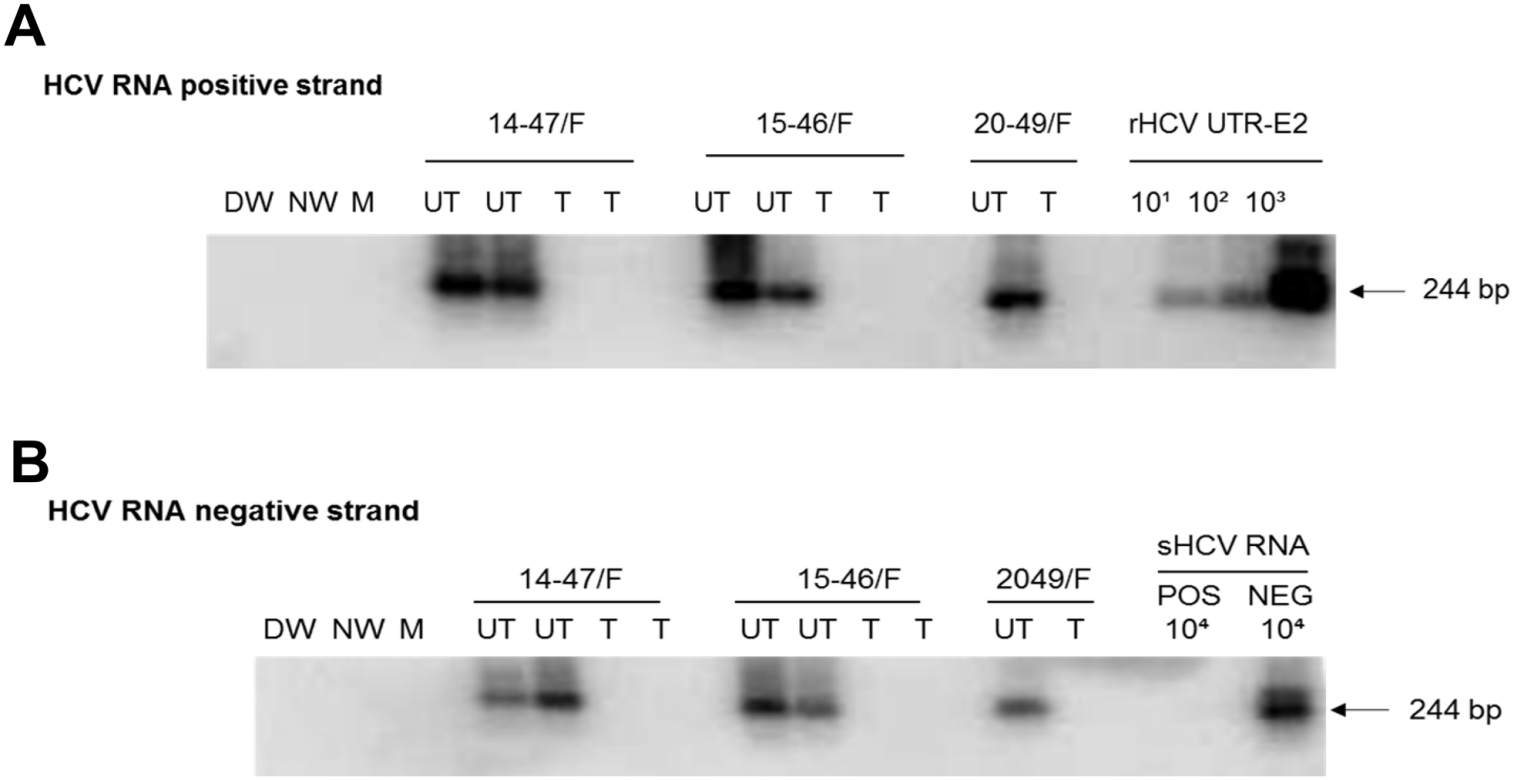
Inhibition of HCV infection in PBMC expressing initially both positive and negative strand of HCV RNA from individuals with a past spontaneous resolution of hepatitis C. PBMC naturally infected with HCV derived from asymptomatic persons followed for 7.4 (14-47/F), 16.1 (15-46/F) or 20.6 (20-49/F) years were treated (T) with 4 μM of TLV or left untreated (UT) in culture for 72 h. Except 20-49/F PBMC, the experiment was performed in duplicate. (A) HCV RNA positive strand was detected by RT-PCR with 5’-UTR-specific primers and amplicon specificity verified by NAH. (B) Virus negative (replicative) strand was identified by the strand-specific RT-PCR/NAH in which synthetic HCV RNA positive (pos) and negative (neg) strands at 10^4^ copies/reaction were used as specificity controls. Other controls were as described in the legend to Fig 2.

Treatment of PBMC reactive for HCV RNA positive and negative strands with TLP completely inhibited HCV expression in PBMC (Fig 5). Thus, PBMC from 14-47/F, 15-46/F and 20-49/F acquired during the second sample collection at 7.4, 16.1 and 20.7 years of follow-up, respectively, totally aborted expression of HCV RNA negative and positive strands after exposure to TLP, while both virus RNA strands remained detectable in control cells treated with 0.5% DMSO alone.

## Discussion

The current study documented persistent traces of HCV in more than half (70.8%) of patients with self-limited HCV without viraemia by conventional tests when assessed by more stringent criteria. HCV carriage was evident after 6.4 and 12.7 years. HCV was identified with similar frequency in plasma (51.3%) and circulating lymphoid cells (51.1%). Examining RNA from very large amounts of plasma (~5 ml) and using PBMC stimulated *ex vivo* improved HCV detection rates, as observed before for SVR after IFN-based therapy [6,12,13,17]. The study also validates a detection approach permitting for greater uniformity and sensitivity in identification of OCI. This approach is based on cumulative experience gained during several previous studies [3,12,13,17,18]. In general, HCV detection in OCI utilizes RNA prepared from large volumes of plasma and intact or mitogen-treated PBMC, testing of serial samples of plasma and circulating lymphoid cells, and detection of virus genome by assays with the greatest sensitivity possible [reviewed in 6].

After spontaneous resolution of HCV infection virus-directed CD4+ and CD8+ T lymphocyte reactivity persists for decades, while humoral responses decline with time [24–26], findings consistent with continuous stimulation of the immune system by low-level HCV replication and trace antigen production. This may be analogous to other asymptomatic carrier states following symptomatic, or unapparent, viral infection such as occult hepadnaviral persistence in humans and woodchucks after resolution of self-limited acute hepatitis or infection with very low amounts of pathogenic virus [27–29].

The rate of detecting HCV RNA in the current study was comparable or lower than that reported in two previous studies using assays with similar sensitivity. In one study, 5 (71.4%) of 7 patients followed up to 5 years after transient hepatitis C were identified as HCV reactive in plasma and/or PBMC, while the HCV RNA replicative strand was detected in 2 of 4 PBMC carrying HCV RNA positive strand [15]. In our study that originally reported on OCI [3], all 5 individuals followed for up to 41 years (mean 21.2 years) carried HCV traces when a single pair of plasma and PBMC was assessed by the methods used in the present work. Thus, 3 of the patients carried HCV both in plasma and PBMC, while two remaining in one of these two compartments. HCV replication was evident in 2 of 3 HCV-reactive PBMC available for the negative strand evaluation [3]. Considering the number of patients investigated, the current study should reflect most closely the actual incidence of OCI long after spontaneously resolution of HCV. Since the similar methods to enhance HCV detection were used in all these studies, a meta-analysis of all 36 patients investigated indicate that 72.2% (n = 26) were HCV RNA trace positive when paired plasma and PBMC were analyzed. In the present investigation, testing a second pair of plasma and PBMC samples a further 4.5 to 7.2 years later confirmed OCI in 5 (55.5%). HCV was not detected in 2 (22.2%) of 9 individuals tested. Others showed a variable pattern (Table 3). Some individuals clear OCI but in others the virus fluctuates at borderline detection levels for years.

In the current study, approximately half of PBMC samples acquired at the mean follow-up time of 5.7 years or 13.1 years carried the HCV RNA positive strand. The majority (11/16; 68.7%) of the positive strand reactive cells were also negative strand positive (Table 2), implying active virus replication. These results are similar to those in two studies in which PBMC from 4 (51.7%) of 7 individuals demonstrated HCV replication for up to 12 years [3,15]. For comparison, the cumulative rate of cases with replicating virus in PBMC during OCI after clinically successful IFN-based therapy followed for up 6 years was 44.2% (46/104 individuals with OCI) [3,9,12,13,15,16,18,30]. In this context, different subsets of PBMC-derived immune cells have been found to carry HCV, its RNA replication strand, intracellular non-structural protein 5a and virus sequence variants not harboured in patients’ plasma or liver [4,18,19,31,32]. Cultured PBMC from individuals with OCI after successful antiviral therapy released HCV virion-like particles identified by immunoelectron microscopy with anti-HCV E2 antibodies and transmitted infection to virus-naïve human primary T cells [9]. The accumulated evidence indicates that HCV residing in immune cells retains biological competence, including infectivity [9,14,33]. Consequently, the detection of HCV positive and, in several cases, replicative strands in PBMC in the current study is indicative that both PBMC and hepatocytes are sites of HCV replication and viral assembly.

Persistence of small quantities of HCV years after spontaneous resolution of HCV has several important aspects. Virologically, the long-term persistence of minute amounts of HCV is not surprising since silent carriage is common for non-cytopathic viral infection that cause chronic or clinically transient infections [27,34]. At this stage, there are insufficient data to comment on the long-term clinical consequences of OCI after apparent spontaneous resolution of HCV. In the current study, mild to severe fibrosis was found on biopsy in 3/8 with mean follow-up 7.1 years and minimal fibrosis in 4 others. In addition, fibroscan results indicative of mild fibrosis was seen in one patient after 14.1 years of observation (Table 1). This is consistent with fibrosis and protracted low grade liver inflammation in some patients with OCI despite clinical SVR 10 years previously [4,5,35]. Further, HCC still develops in 3.9% despite SVR after IFN-based therapy [36–38] and OCI after spontaneous resolution of HCV may retain similar potential. Whether OCI could act as a “second hit” promoting hepatic injury in individuals with non-alcoholic fatty liver disease (NAFLD) or alcohol-related liver disease remains almost completely unexplored.

As this study showed, spontaneous resolution of hepatitis C does not always coincide with molecular eradication of HCV and an established asymptomatic infection can persist for decades. This is new information that modifies our current understanding of the natural history of HCV infection. It also draws attention to issues potentially important to the pathogenicity and epidemiology of asymptomatic HCV infection that is undetectable by conventional testing, *i*.*e*., a possibility of long-term pathological consequences and subclinical transmission of virus. Overall, the results of our study imply that the long-term morbidity of asymptomatic HCV carriage should be examined even in individuals who achieve undetectable HCV after self-resolved hepatitis C and their need for anti-HCV treatment should be assessed.

## Supporting Information

S1 Table Sequences of oligonucleotide primers used to amplify HCV genome fragments. (PPTX)
