# Supplementary material for: Persistence of Hepatitis C Virus Traces after Spontaneous Resolution of Hepatitis C
Source: PLoS One. 2015 Oct 16;10(10):e0140312. doi: 10.1371/journal.pone.0140312 (PMC4608821; doi:10.1371/journal.pone.0140312)
Supplement: S1 Table — (PPTX) [file pone.0140312.s001.pptx]

## Slide 1
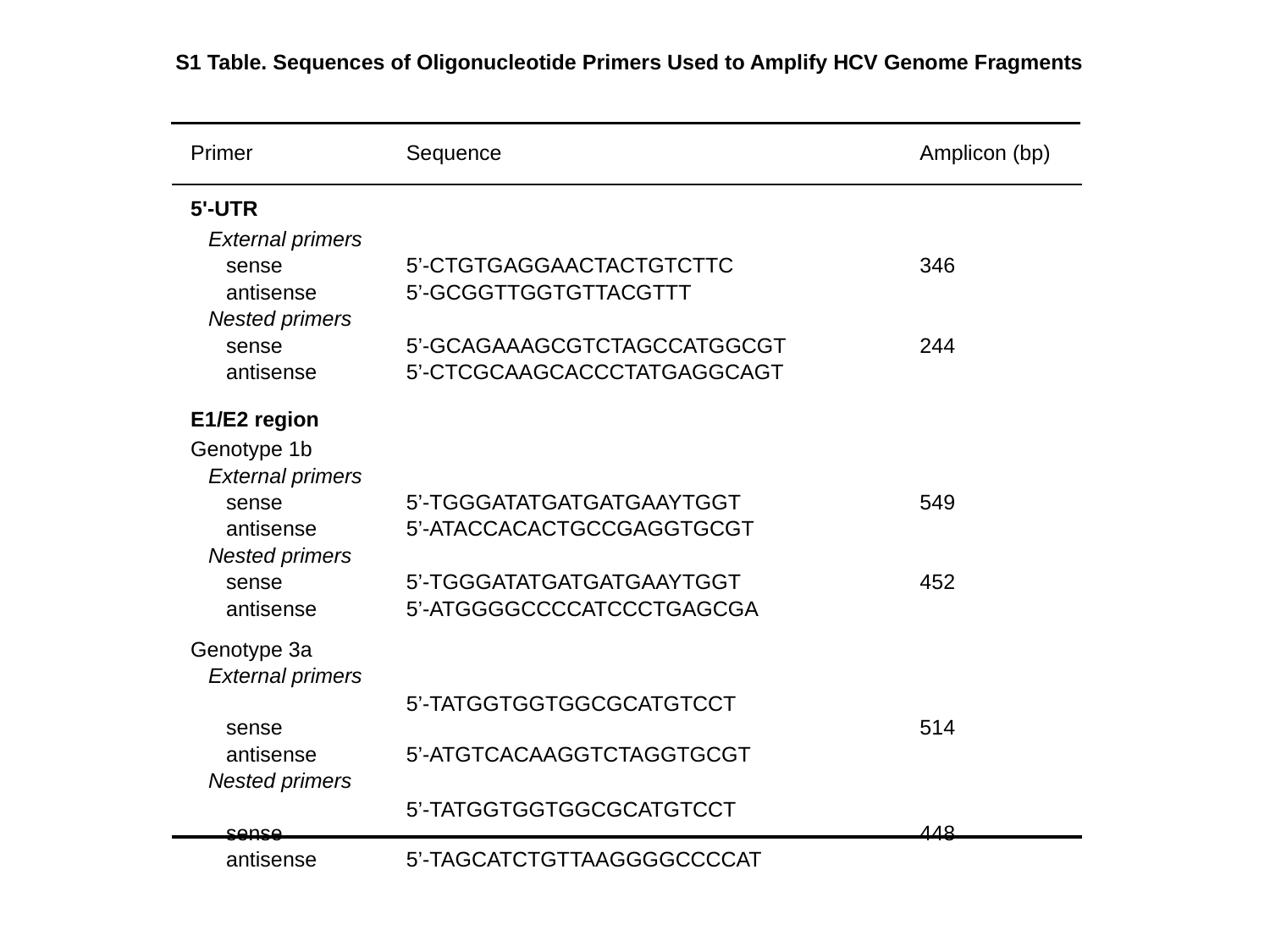

S1 Table. Sequences of Oligonucleotide Primers Used to Amplify HCV Genome Fragments
| Primer | Sequence | Amplicon (bp) |
| --- | --- | --- |
| 5ʹ-UTR | | |
| External primers | | |
| sense | 5’-CTGTGAGGAACTACTGTCTTC | 346 |
| antisense | 5’-GCGGTTGGTGTTACGTTT | |
| Nested primers | | |
| sense | 5’-GCAGAAAGCGTCTAGCCATGGCGT | 244 |
| antisense | 5’-CTCGCAAGCACCCTATGAGGCAGT | |
| E1/E2 region | | |
| Genotype 1b | | |
| External primers | | |
| sense | 5’-TGGGATATGATGATGAAYTGGT | 549 |
| antisense | 5’-ATACCACACTGCCGAGGTGCGT | |
| Nested primers | | |
| sense | 5’-TGGGATATGATGATGAAYTGGT | 452 |
| antisense | 5’-ATGGGGCCCCATCCCTGAGCGA | |
| Genotype 3a | | |
| External primers | | |
| sense | 5’-TATGGTGGTGGCGCATGTCCT | 514 |
| antisense | 5’-ATGTCACAAGGTCTAGGTGCGT | |
| Nested primers | | |
| sense | 5’-TATGGTGGTGGCGCATGTCCT | 448 |
| antisense | 5’-TAGCATCTGTTAAGGGGCCCCAT | |
